# Exploratory miRNA profiling from serum and bone tissue of mice with T1D-induced bone loss

**DOI:** 10.3389/fendo.2024.1477257

**Published:** 2024-12-24

**Authors:** Souad Daamouch, Andreas Diendorfer, Matthias Hackl, Gabriele Christoffel, Lorenz C. Hofbauer, Martina Rauner

**Affiliations:** ^1^ Department of Medicine III and Center for Healthy Aging, Technische Universität Dresden, Dresden, Germany; ^2^ TAmiRNA, Vienna, Austria; ^3^ Qiagen, Hilden, Germany

**Keywords:** miRNAs, T1D, serum, bone, NGS, RT-PCR

## Abstract

Type 1 diabetes (T1D) represents a significant health burden worldwide, with associated complications including bone fragility. Current clinical methods and biomarkers for assessing bone health and predicting fracture risk in T1D are limited and lack accuracy. MicroRNAs (miRNAs) have emerged as potential biomarkers for predicting T1D-induced bone loss, although comprehensive profiling studies are lacking. Previous investigations have indicated a link between dysregulated miRNA expression levels and impaired bone health in T1D. Therefore, in this study, we explored differential miRNA expression levels in serum and bone tissue of mice with T1D-induced bone loss using Next Generation Sequencing (NGS). T1D was induced using streptozotocin in male wild-type mice. Serum and bone tissues were analyzed at 14 weeks of age, following the prior characterization of bone loss in this mouse model. MiRNA profiling was conducted using two-independent NGS analyses and validated through quantitative RT-PCR. NGS profiling identified differential expression of miRNAs in serum and bone tissue of T1D mice compared to controls. The first NGS analysis revealed 24 differentially expressed miRNAs in serum and 13 in bone tissue. Especially, miR-136-3p was consistently downregulated in both serum and bone tissue. However, the second NGS analysis presented a distinct set of dysregulated miRNAs, with miR-206-3p overlapping in both tissues but exhibiting differential expression patterns. Surprisingly, miR-144-5p, miR-19a-3p, and miR-21a-5p displayed contrasting regulatory patterns between NGS and qPCR analyses. Finally, gene network analysis identified associations between dysregulated miRNAs and pathways involved in bone physiology, including TGF-beta, PI3-Akt signaling, and osteoclast differentiation in humans. In conclusion, our study offers initial insights into dysregulated miRNAs associated with T1D-induced bone loss, but also highlights the lack of consistency in the results obtained from miRNA sequencing in different cohorts. Thus, further investigation is needed to better understand the complexities of miRNA analyses before they can be established as reproducible biomarkers for predicting bone health in T1D.

## 1 Introduction

Type 1 diabetes (T1D) is a chronic condition triggered by an autoimmune response that hinders the body’s natural regulation of blood glucose levels. While this disease is frequently diagnosed in childhood, with 80% of beta cells typically already destroyed by that time (1, 2), the International Diabetes Federation reported in 2022 that 64% of individuals with T1D fall within the 20-59 age group. Thus, not only the incidence of T1D is increasing worldwide, but also the proportion of older individuals suffering from T1D. Due to the chronic nature of the disease, long-term T1D is associated with several complications that impact human health, including cardiomyopathy (3, 4), nephropathy (5, 6), and bone fragility (7, 8). Individuals with T1D have a six-fold higher risk of hip fractures compared to the general population, highlighting the need for a better understanding of the underlying pathomechanisms of bone fragility as well as the development of better prediction tools to assess patients at risk for fracture (9, 10).

Current clinical methods employed to assess bone fragility in T1D, such as bone turnover markers (BTMs), dual-energy X-ray absorptiometry (DXA), the fracture risk assessment tool (FRAX), and trabecular bone score (TBS) fall short in predicting fracture risk and present various limitations. Given the global burden of T1D and diabetic osteoporosis, there is an urgent need for affordable and innovative methods to overcome current limitations and more accurately predict fracture risk in T1D. Recent investigations have provided promising insights into the potential use of miRNAs. MiRNAs, small non-coding RNAs with a length of 19-22 nucleotides, play a crucial role as post-transcriptional gene regulators (11, 12). While limited studies have explored the role of miRNAs in bone diseases associated with T1D (13), evidence of dysregulated miRNAs, such as miR-148a-3p and miR-21-5p has been reported, showing a negative correlation with bone mineral density (BMD) in the serum of 15 patients with T1D compared to 14 matched non-diabetic subjects (14). In another study, miRNA expression patterns were assessed in 58 diabetic rats and 58 non-diabetic rats during the fracture healing process on days 5 and 11 within femurs. This study observed the dysregulation of five miRNAs (miR-140-3p, miR-140-5p, miR-181a-1-3p, miR-210-3p, and miR-222-3p), which were previously reported as dysregulated in the context of impaired fracture healing in diabetic rats (15). These findings provide evidence that specific miRNAs are dysregulated in T1D and highlight miRNAs as potential biomarkers for bone fragility in T1D. To date, only pre-defined panels of miRNAs have been studied in the context of T1D bone disease. However, untargeted approaches to assess the regulation of miRNAs in total in T1D are warranted and may be beneficial to identify novel miRNA targets or miRNA signatures that may serve as novel biomarkers to enhance fracture risk assessment in T1D.

The aim of this study was to perform an unbiased miRNA profiling approach from bone tissue and serum of mice with T1D-induced bone loss to assess local *vs*. systemic miRNA regulations using Next Generation Sequencing (NGS). A second aim was to assess the reproducibility of these findings in two independent mouse cohorts. To achieve this, we conducted two independent miRNA profiling analyses on bone and serum samples, employing two independent mouse cohorts and two different library preparation methods. Our investigations revealed first, minor overlap between miRNAs regulated in bone tissue vs. serum and second, variability in dysregulated miRNAs between both NGS profiling experiments. Thus, when evaluating miRNAs or miRNA signatures resulting from NGS experiments as potential biomarkers for disease prediction or monitoring, validation procedures are clearly needed before they can be applied as biomarkers.

## 2 Materials and methods

### 2.1 STZ-induced T1D mouse model

In order to establish the T1D mouse model, we administered streptozotocin (STZ) via intra-peritoneal injections at a dose of 45 mg/kg to 10-week-old male C57BL/6J mice. This treatment was repeated for five consecutive days. The control group received injections with citrate buffer (CB) (16). One week after the STZ or CB injections, we assessed blood glucose levels obtained from the tail vein. The onset of diabetes was defined with blood glucose levels reaching 250 mg/dl. Over the course of the study, we monitored blood glucose levels and body weight on a weekly basis, up to week 14, at which point all the mice were euthanized. To ensure uniform conditions, all mice were given standard diets and had continuous access to water. They were housed in groups of four or five per cage, maintained at room temperature, and adhered to a 12-hour light/dark cycle. Ethical approval for all procedures involving mice was obtained from the Landesdirektion Sachsen (TVV 18/2020) and the institutional animal care committee.

### 2.2 Glucose tolerance test

To assess the glycemic state of T1D mice, a glucose tolerance test (GTT) was carried out following an overnight fasting period. Blood glucose levels were assessed at intervals of 15, 30, 60, 90, and 180 minutes after intraperitoneal administration of a 2 g/l glucose solution, utilizing a glucometer (ACCU CHEK Aviva III; Roche Diabetes Care, Mannheim, Germany).

### 2.3 Bone microarchitecture analysis

We employed micro-computed tomography (microCT) to assess the bone mass and microarchitecture of the distal femur. The microCT scans were conducted ex vivo using a vivaCT 40 scanner manufactured by Scanco Medical in Brüttisellen, Switzerland. These scans were performed with an energy level of 70 kVp with a resolution of 10.5 µm isotropic voxel size (utilizing 114 mA and an integration time of 200 msec). A total of one hundred slices from the distal femur were subjected to analysis using Scanco Medical’s standard protocols. This analysis was carried out to assess trabecular bone parameters, which include bone trabecular thickness (Tb.Th) and trabecular bone mineral density (Tb. BMD). Additional MicroCT parameters were assessed in our previous study (17).

### 2.4 MiRNA isolation, cDNA synthesis and quantitative RT-PCR

The extraction of total RNA from bone tissue was carried out using TRIzol reagent (Invitrogen, Darmstadt, Germany) in accordance with the provided manufacturer’s guidelines. Subsequently, the RNA concentration was assessed using a Nanodrop ND-1000 spectrophotometer (Thermo Scientific/PEQLAB, Erlangen, Germany). Post-mortem, the bones were collected and flushed out with PBS before the RNA extraction process. For miRNA reverse transcription, 5 nanograms of total RNA were employed and reverse-transcribed using the miRCURY LNA RT Kit (#339340; Qiagen, Hilden, Germany). Following this step, the resulting cDNA samples were diluted at a 1:40 ratio. Quantitative real-time PCR was performed using miRCURY LNA SYBR Green (#339345; Qiagen, Hilden, Germany). The PCR conditions consisted of an initial denaturation at 95°C for 2 minutes, followed by denaturation at 95°C for 10 seconds, and annealing/extension at 56°C for 60 seconds, repeated for 40 cycles. To normalize the data, the miRNA expression levels were compared to those of the 5S housekeeping gene, employing the ΔΔCT method, and the results are presented as fold changes (x-fold). All procedures adhered to the manufacturer’s instructions.

### 2.5 NGS miRNA profiling using Illumina sequencing

We performed total RNA extraction from bone tissue of T1D and control mice and subsequently employed the RealSeq-Biofluids Plasma/Serum miRNA Library kit for Illumina sequencing (RealSeq Biosciences) in accordance with the manufacturer’s protocol (17). In this study, two independent NGS analyses were conducted on bone and serum samples from both T1D and non-diabetic mice. The purpose was to evaluate the reproducibility of the findings.

Prior to library preparation, we assessed RNA quality using the Agilent Bioanalyzer DNA 1000 kit and associated reagents (Agilent Technologies, Waldbronn, Germany). Only samples with a RIN over 6 were taken for further analyses. The NGS Illumina sequencing process is based on the “sequencing by synthesis” method, which aims to produce sequence reads of approximately 32-40 base pairs by simultaneously sequencing tens of millions of surface-amplified DNA fragments (18). The library preparation involves a sequence of six consecutive steps: adapter ligation, surface attachment, bridge amplification, denaturation, clustering, and single base extension.

In our study, two library preparation protocols were employed as per institutional methods. Serum and bone samples RNA underwent library preparations using RealSeq Biosciences and QIAseq miRNA Library Kit following manufacturer protocols, with 8.5 μl and 4 μl RNA input, respectively. Amplification steps comprised 18-23 cycles. Initial library quality control utilized the Agilent DNA 1000 kit to evaluate fragment distribution. Subsequently, libraries were equimolarly pooled and subjected to size-selection using the BluePippin system with a 3% agarose cassette (100-250 kb) to eliminate DNA fragments outside the desired range. A second library quality check was performed using the Agilent high sensitivity DNA kit. Finally, libraries were sequenced on an Illumina NextSeq550, following manufacturer instructions.

### 2.6 Statistical analysis

Data are given as mean ± SD. A two-sided, unpaired Student’s t test was used in statistical analyses to compare two groups. Graphs were generated using GraphPad Prism 9.0 (GraphPad, La Jolla, CA, USA). Statistical significance was defined as a P value of less than 0.05. The analysis of RNA-Seq data was conducted using MiND, a software package for data analysis that produces comprehensive QC data, unsupervised clustering analysis, normalized miRNA count matrices, and differential expression analysis from raw NGS data (19, 20). Assessment of the overall quality of next-generation sequencing data involved both automatic and manual evaluations using fastQC v0.11.9 and multiQC v1.10.

To increase robustness of the false discovery rate (FDR) correction and eliminate low-abundance miRNAs, the independent filtering method of DESeq2 was modified for compatibility with edgeR. Volcano plots were employed to visually represent the relationship between logFC and the statistical significance of observed miRNA level changes. Consequently, miRNAs displaying significant differential expression with an FDR below 0.05 are highlighted in green.

## 3 Results

### 3.1 MiRNA profile in serum and bone tissue of T1D mice with bone loss

#### 3.1.1 Characterization of STZ-induced T1D and bone loss in male mice

We used the well-established STZ-induced T1D mouse model and characterized its metabolic and bone phenotype at 14 weeks of age, 4 weeks after T1D induction. The results were reported elsewhere (17). Briefly, and consistent with previous studies (21, 22), we observed a significant decrease in the body weight, impaired glucose tolerance, and bone loss with low bone formation and high bone resorption in T1D compared to non-diabetic mice in both cohorts. Thus, this T1D mouse model exhibited a consistent and validated profile for further investigations. To provide additional characterization, we assessed several metabolic and bone parameters at the end of the experiment (14 weeks of age). A detailed summary of these findings is presented in our previous manuscript (17). These results highlight the significant metabolic and skeletal alterations induced by T1D, supporting the robustness of this model.

#### 3.1.2 NGS volcano plots of serum and bone

We next performed an exploratory miRNA profiling from serum and femoral bone samples collected from T1D versus control mice. Therefore, we conducted two separate NGS analyses, each using three samples per tissue type (serum and femur) from T1D and non-diabetic mice.

**Figure 1:**
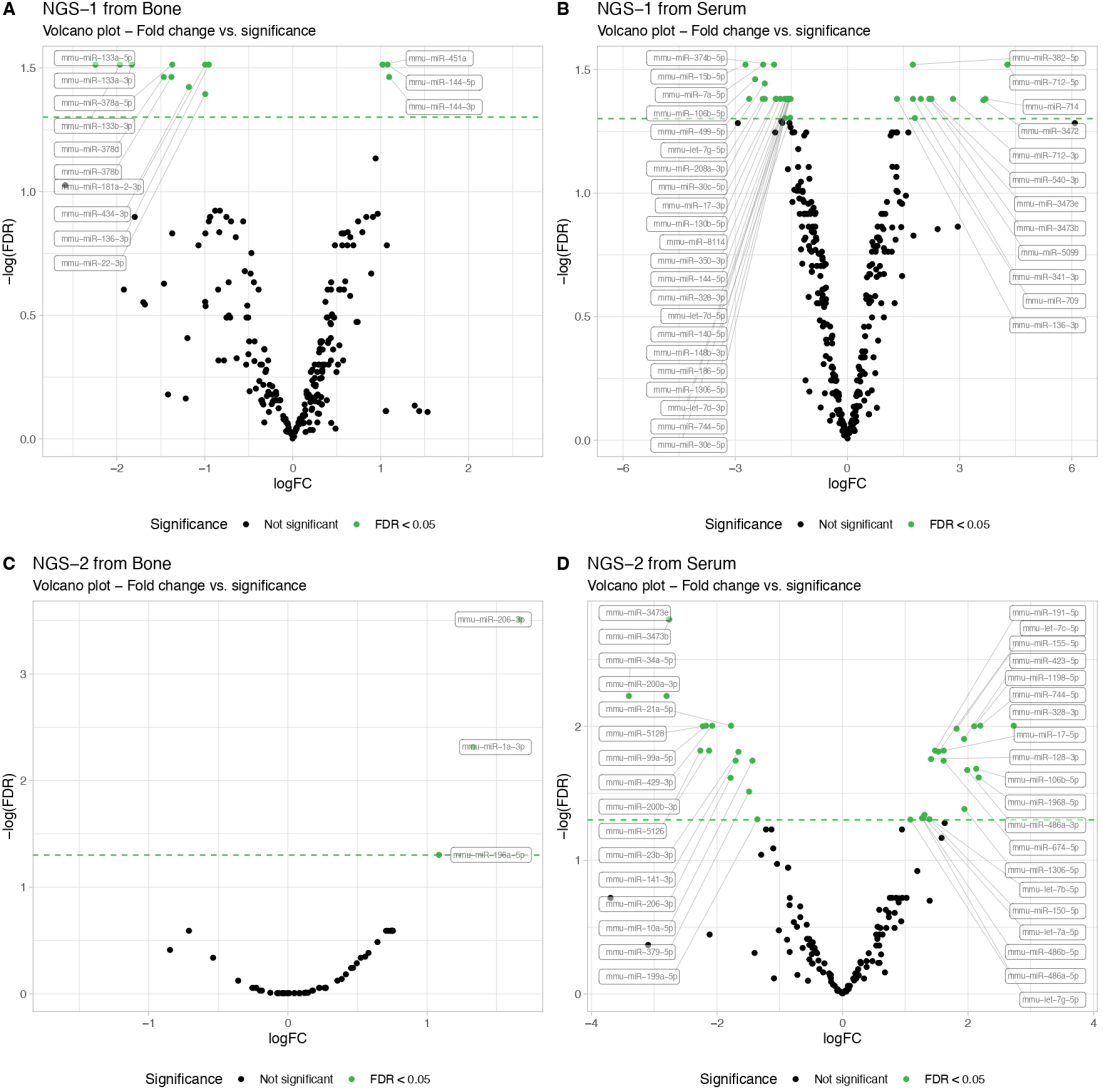
Next-Generation Sequencing (NGS) miRNA profiling in serum and femur bone from mice with T1D compared to non-diabetic mice. The NGS data results from exploratory miRNA profiling conducted on serum (N=3 per group) and femur bone (N=3 per group) of STZ-induced T1D versus non-diabetic mice, revealing differentially expressed miRNAs. Volcano plots illustrate the NGS miRNA data profiling from the first analysis, showing **(A)** serum and **(B)** bone mice samples. A second NGS analysis, performed in another independent cohort, similarly showcases **(C)** serum and **(D)** bone mice samples. MiRNA expression levels are indicated by the logarithm of fold change (logFC). All data are statistically significant with a False Discovery Rate (FDR) of less than 0.05.

In the first NGS analysis, we identified 24 differentially expressed miRNAs in serum samples, in contrast to 13 in bone tissue. Specifically, in bone tissue, we observed 3 upregulated miRNAs (miR-144-3p, miR-144-5p and miR-451a) and 10 downregulated miRNAs (miR-133a-5p, miR-133b-3p, miR-133a-3p, miR-378d, miR-378b, miR-378a-5p, miR-181a-2-3p, miR-434-3p, miR-22-3p and miR-136-3p) (**Figure 1A** NGS-1 from bone). In serum samples, we found 12 upregulated and 12 downregulated miRNAs when comparing T1D and control mice (**Figure 1B** NGS-1 from serum).

Intriguingly, the second NGS analysis conducted in another independent cohort, presented a different set of dysregulated miRNAs. While bone tissue displayed 3 up-regulated miRNAs (miR-206-3p, miR-1a-3p and miR-196a-5p) when comparing T1D and non-diabetic mice (**Figure 1C** NGS-2 from bone), serum data exhibited 12 up- and 12 down-regulated miRNAs (**Figure 1D** NGS-2 from serum), None of the up- or down-regulated miRNAs overlapped with the results from the first NGS analysis.

#### 3.1.3 VENN diagrams from NGS data showing overlapping miRNAs in serum and bone

**Figure 2:**
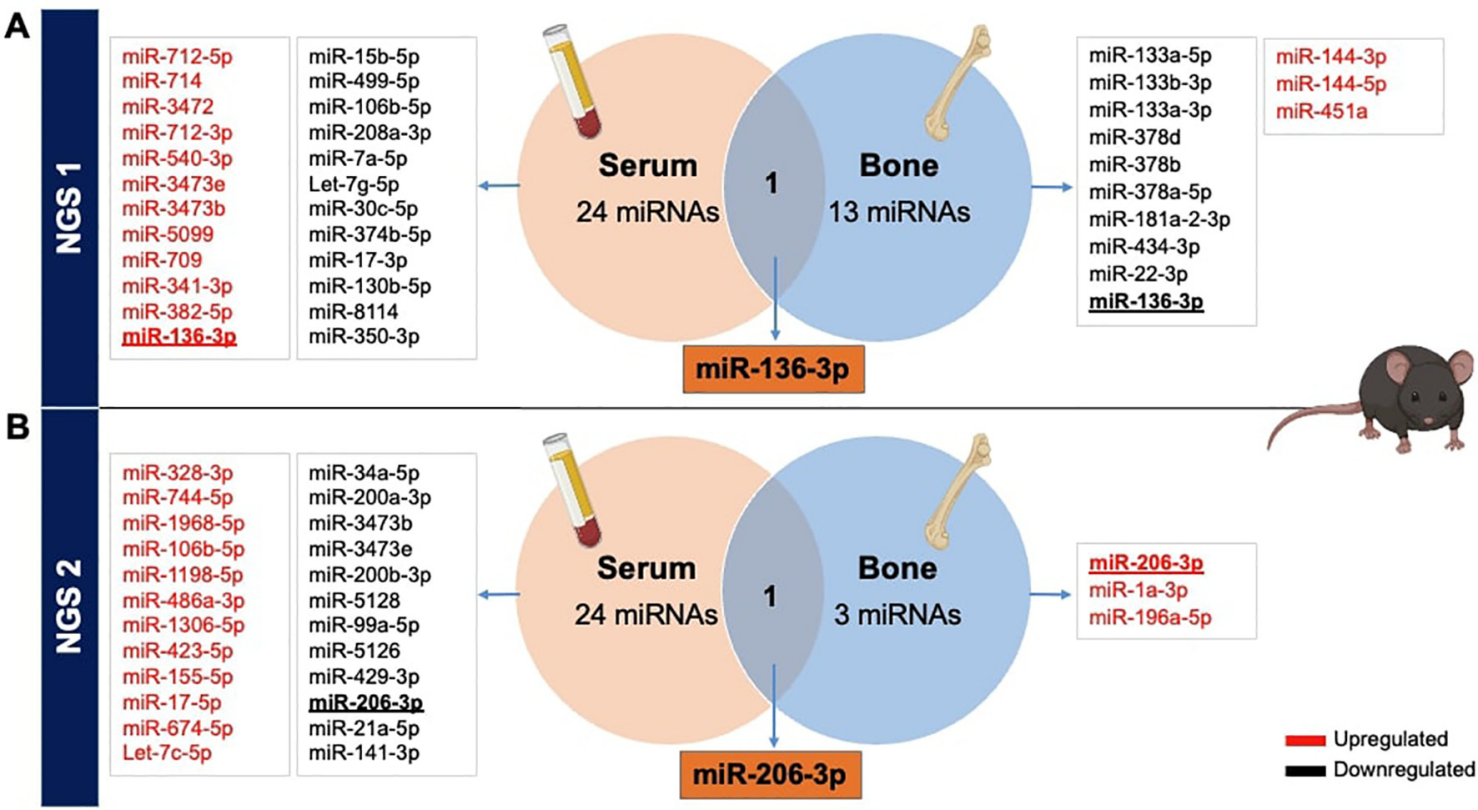
Overlapping miRNAs from NGS in serum and bone tissues of STZ-induced T1D versus non-diabetic mice. Identification of differentially expressed miRNAs obtained from serum and femoral bones, from 2 independent NGS exploratory miRNA profiling, reported in VENN diagrams. **(A)** The primary NGS detected miR-136-3p as a commonly dysregulated miRNA, while **(B)** the second NGS identified miR-206-3p between both tissues of T1DM versus non-diabetic mice.

Afterwards, to ascertain potential overlaps of miRNAs between serum and bone tissue using NGS data, we generated Venn diagrams based on both NGS analyses. It became evident that there were no shared miRNAs between the two NGS datasets. Interestingly, the initial NGS evaluation revealed that miR-136-3p was upregulated in serum while downregulated in bone tissue (**Figure 2A**). Additionally, in the second NGS analysis, a distinct miRNA, miR-206-3p, was found to overlap in both tissues but exhibited again a differential expression pattern. It was downregulated in serum but upregulated in bone tissue of T1D mice (**Figure 2B**).

### 3.2 Discrepant miRNA expression level measured between NGS and qPCR in bone tissue

**Figure 3:**
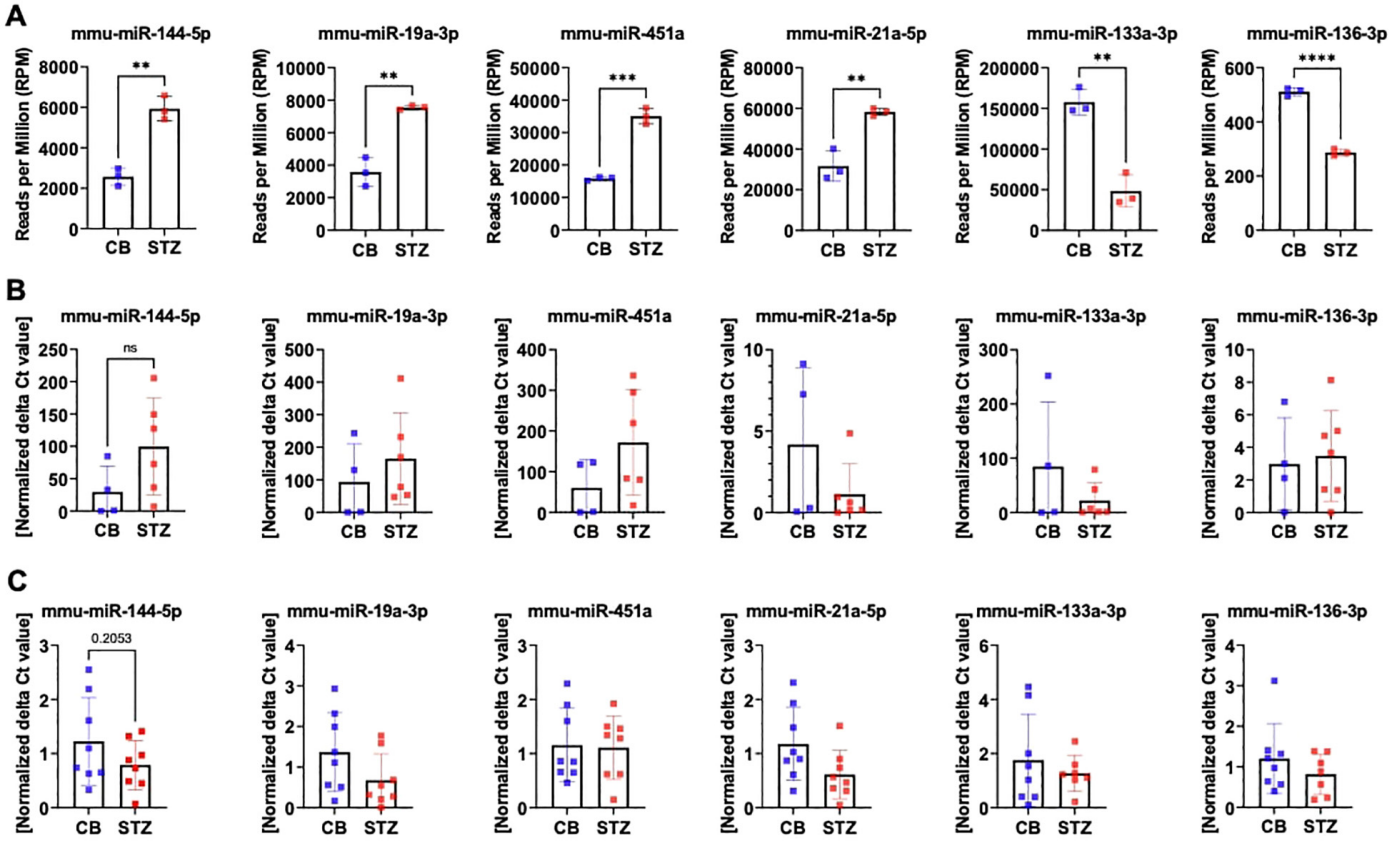
MiRNA expression level assessed by RT-PCR in mouse bone tissues. Quantification of selected miRNA targets from bone tissue of 14-week-old T1D versus non-diabetic mice was performed using NGS and RT-PCR based on the 1^st^ NGS analysis. Panel **(A)** showcases NGS data depicting miRNA expression levels in reads per million (RPM). Panel **(B)** indicates miRNA expression levels assessed in the identical bone samples employed for NGS, while panel **(C)** illustrates miRNA expression levels assessed in bone samples from a separate cohort by RT-PCR. Data are indicated as mean ± SD (N=3-8). A Student's t-test was used for statistical analysis, and significant results were denoted as follows: **p<0.01, ***p<0.001.

The discrepancy in miRNA expression levels between serum and bone tissue underscores that tissue-based differences in miRNA transcription are not necessarily mirrored by changes in circulating miRNA levels. Given our primary interest in bone tissue, we exclusively validated miRNA candidates through further qPCR analysis using bone samples in this study. For the NGS measurements presented in panel A, only the highest-quality RNA samples (n=3 per group) were selected, based on stringent quality criteria such as a RIN between 6 and 10, as measured by the bioanalyzer. This careful selection ensured the reliability and robustness of the sequencing data by minimizing variability due to RNA integrity. Additionally, to date, no studies have directly investigated miRNA expression in bone tissue related to T1D. Thus, we proceeded with the selection of a set of miRNAs, based on our initial NGS analysis of bone tissue samples and the existing literature. The following miRNAs were chosen for additional qPCR validation: miR-144-5p, miR-19a-3p, miR-451a, miR-21a-5p, miR-133a-3p, and miR-136-3p (**Figure 3**). To provide an overview of their expression patterns from NGS analysis, the normalized reads per million (RPM) for these miRNAs are shown in panel A (**Figure 3A**). We then evaluated these miRNAs using two different approaches to validate and ensure the reproducibility of identical targets through qPCR. First, we measured miRNA levels using the same samples employed in our initial NGS analysis (**Figure 3B**). Secondly, we measured miRNA levels from another independent cohort of T1D mice (**Figure 3C**).

By using the same set of bone samples utilized in the NGS analysis, qPCR analysis confirmed a trend toward elevated levels for miR-144-5p, miR-19a-3p, and miR-451a. Similarly, miR-133a-3p displayed a declining trend, though it did not achieve statistical significance. However, miR-21a-5p exhibited contrasting regulatory patterns, while miR-136-3p, previously identified as downregulated, displayed no significant change by qPCR (**Figure 3B**).

Subsequent qPCR analysis using a distinct set of bone samples revealed differing outcomes concerning miRNA expression. Particularly, miR-144-5p, miR-19a-3p, and miR-21a-5p demonstrated contrasting trends in regulation. However, the downregulation patterns of miR-451a, miR-133a-3p, and miR-136-3p remained consistent with the NGS findings (**Figure 3C**).

### 3.3 Tissue-type enrichment analysis of dysregulated miRNAs

To evaluate the expression levels of these miRNA candidates under steady conditions in humans, we conducted a mapping of the top 9 highly enriched tissues for each of our six miRNAs. Additionally, we included bone tissue as our primary focus, even if it did not consistently rank among the top nine enriched tissues. These data were obtained from the miRNA Tissue Atlas database (23).

**Figure 4:**
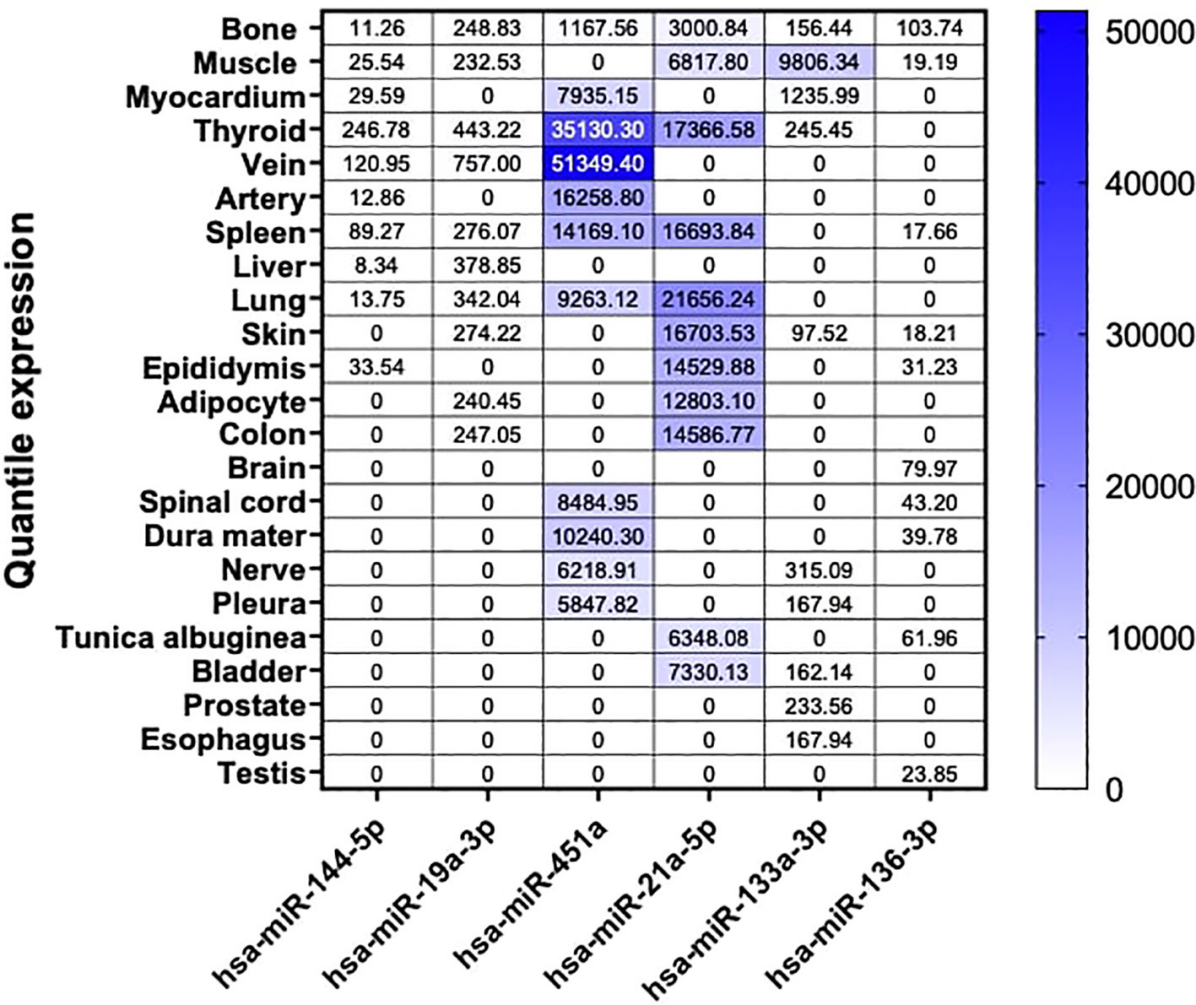
Tissue-specific miRNA enrichment analysis in Human. Custom heatmap representing an overview of bone and the top 9 highly enriched tissue from our 6 selected miRNA candidates in Human. Bone tissue is presented in this heatmap as our tissue of interest. These results are expressed in normalized quantile for each miRNA respectively. These data are extracted from the miRNA Tissue Atlas database.

Our findings revealed that our six miRNA targets displayed differential abundance in human tissues, with the highest expression levels observed for hsa-miR-451a, hsa-miR-21a-5p, and hsa-miR-133a-3p. Interestingly, despite the dysregulation of these miRNAs observed in our NGS analysis in mouse bone tissue, they exhibited low expression in human bone. Among our three highly abundant miRNAs, we observed enriched expression in tissues such as veins, thyroid, arteries, spleen, lungs, muscles, skin, and adipocytes. Moreover, for the less abundant miRNAs, hsa-miR-144-5p was predominantly expressed in the thyroid, and hsa-miR-19a-3p in the veins. Interestingly, although generally low in expression, hsa-miR-136-3p was predominantly expressed in human bone tissue (**Figure 4**).

### 3.4 Gene network and potential signaling pathways involved in bone pathophysiology triggered by dysregulated miRNAs

To investigate the gene and pathway networks associated with these miRNA targets, we used the miRPathDB v2.0 database, which is based on the KEGG database of humans. We identified significant signaling pathways that are targeted by the dysregulated miRNAs and may be involved in bone metabolism.

Our analysis revealed that the majority of our dysregulated miRNAs and their targeted genes are prominently associated with TGF-beta, PI3-Akt signaling, osteoclast differentiation, and adherens junction signaling pathways. Additionally, they exhibited connections to Wnt and TNF signaling pathways, albeit to a lesser extent compared to the previously mentioned pathways. Notably, we identified five genes targeted at least four times within these signaling pathways: SMAD4, MAPK1, MYC, TGFBR2, and AKT1 (**Table 1**). Taken together, this suggests the potential significance of the TGF-beta/BMP and Wnt pathways in the context of bone physiopathology in T1D (**Table 1**).

**Table 1:** Gene network related to miRNA targets in Human.

Pathways	miRNA	Hits	P-val.	Targets
**TGF-beta signaling**	miR-144-5p	3	0.005	ID4, ROCK1, **SMAD4**
	miR-19a-3p	10	0.005	BAMBI, BMPR2, **MAPK1**, **MYC, SMAD4**, SMAD5, **TGFBR2**, THBS1, TNF, ZFYVE9
	miR-451a	2	0.013	**MAPK1**, **MYC**
	miR-21a-5p	9	0.015	BMPR2, GDF5, **MYC**, SMAD7, SP1, TGFB1, TGFB2, **TGFBR2**, ZFYVE16
**p53 signaling**	miR-144-5p	3	0.005	CCNE1, CCNE2, MDM4
	miR-19a-3p	11	6.13e-4	CCND1, CCND2, CHEK1, CHEK2, FAS, PTEN, SESN3, THBS1, TNFRSF10B, TP53, ZMAT3
	miR-21a-5p	10	0.002	APAF1, CASP8, CCNG1, CDK6, FAS, MDM4, PTEN, SERPINB5, SESN1, TNFRSF10B
	miR-133a-3p	5	0.002	CASP9, CDKN1A, IGF1, SESN2, SESN3
**PI3K-Akt signaling**	miR-144-5p	4	0.015	CCNE1, CCNE2, ITGA3, MET
	miR-451a	8	2.89e-6	**AKT1**, BCL2, IKBKB, IL6, IL6R, **MAPK1**, **MYC**, TSC1
	miR-21a-5p	23	0.009	AKT2, ATF2, BCL2, BRCA1, CDK6, COL4A1, COL5A2, EGFR, FASLG, FGF12, FOXO3, GNB4, IGF1R, ITGB8, **MYC**, NFKB1, PDGFD, PIK3R1, PTEN, PTK2, SGK3, TLR4, VEGFA
	miR-133a-3p	11	9.76e-4	ANGPT4, BCL2L1, CASP9, CDKN1A, COL1A1, EGFR, IGF1, IGF1R, MCL1, NGFR, THBS2
**Wnt signaling**	miR-144-5p	2	0.028	ROCK2, **SMAD4**
	miR-19a-3p	12	0.020	BAMBI, CCND1, CCND2, CSNK2A1, FZD6, **MYC**, PRICKLE2, PRKACB, **SMAD4**, TP53, WNT10A, WNT7B
**Osteoclast differentiation**	miR-19a-3p	11	0.020	**AKT1**, MAP3K14, **MAPK1**, MAPK14, PIK3CA, PIK3R3, SOCS1, SOCS3, **TGFBR2**, TNF, TNFRSF11A
	miR-451a	3	0.003	**AKT1**, IKBKB, **MAPK1**
**Regulation of actin cytoskeleton**	miR-144-5p	3	0.023	ITGA3, ROCK1, ROCK2
**Calcium signaling**	miR-133a-3p	5	0.031	CACNA1C, EGFR, ERBB2, ITPKB, PDE1A
**TNF signaling**	miR-451a	5	1.80e-5	**AKT1**, IKBKB, IL6, **MAPK1**, MMP9
	miR-21a-5p	15	1.56e-4	AKT2, ATF2, CASP8, CCL20, CEBPB, CXCL10, DNM1L, FAS, ICAM1, IL1B, JAG1, MAP2K3, MMP9, NFKB1, PIK3R1
**Adherens junction**	miR-144-5p	2	0.016	MET, **SMAD4**
	miR-19a-3p	7	0.039	ACTB, CSNK2A1, **MAPK1**, PTPRB, **SMAD4**, **TGFBR2**, WASL

Identification of significant enriched signaling pathway potentially linked to bone metabolism, using KEGG database based on experimental evidence for the 5 following miRNAs: hsa-miR-144-5p, hsa-miR-19a-3p, hsa-miR-451a, hsa-miR-21a-5p. Genes reported in bold indicate their presence among signaling pathways at least four times. Predicted targets of hsa-miR-136-3p are nor presented here. These data are extracted from miRPathDB v2.0 database.

## 4 Discussion

In this study, an exploratory and unbiased miRNA profiling was conducted on serum and bone samples obtained from STZ-induced T1D male mice, with a comparative analysis against matched non-diabetic mice. The primary objective was to identify dysregulated miRNA targets potentially linked to T1D-induced bone fragility. We hypothesized that by studying bone samples in addition to serum samples from T1D mice, we could find specific miRNA candidates linked to bone fragility in T1D. To reinforce the robustness and significance of our findings, miRNA profiling was carried out on two independent mouse cohorts, which were generated through separate experiments conducted at different time points.

We employed the STZ-induced T1D mouse model, as these mice effectively mimic T1D conditions, displaying notable decreases in body weight, elevated blood glucose levels, and impaired bone mass (17). Thus, we considered this model a well-validated platform for further exploratory miRNA profiling. Studies have previously highlighted the potential of miRNAs as biomarkers for bone fragility (24–26). Importantly, their accessibility in biofluids prevents from invasive biopsy procedures to assess disease progression. Previous research has investigated on the regulation of predefined and specific miRNAs in the circulation and sometimes even in bone biopsies of individuals with diabetic bone disease (14, 27). Nevertheless, no studies have yet conducted unbiased exploratory miRNA profiling to discover novel miRNAs signatures specific to bone tissues.

Our initial miRNA profiling revealed several miRNAs with differential regulation when comparing T1D to non-diabetic mice. This discovery aligns with prior research, contributing to the growing evidence on miRNA dysregulation in T1D (24, 28, 29). The majority of significantly dysregulated miRNAs were identified in serum samples (12 upregulated, 12 downregulated), while fewer were dysregulated in bone tissue. This may be explained by a more restricted portfolio of miRNAs in bone tissue, while in the serum, many tissues contribute to the altered miRNA profile. Across both NGS analyses, only three miRNAs were consistently upregulated in the serum. Setting aside the divergence observed in the two NGS experiments, miR-136-3p emerged as a shared miRNA target in both bone and serum during our initial NGS analysis. This miRNA exhibited downregulation in both tissues of T1D mice and suggests a potentially noteworthy dysregulation within the context of T1D in this particular mouse cohort. On the other hand, our second analysis using NGS uncovered a distinct miRNA intersection with miR-206-3p. Notably, miR-206-3p demonstrated an upregulation within the bone but exhibited downregulation at the systemic level. This finding implies the likelihood of a tissue-specific regulation of miR-206-3p, particularly concerning bone-related processes. Despite its well-documented abundance in muscle and its classification as a myomiR (30), findings from the miRPath miRNA database suggest the involvement of this miRNA in pathways related to bone, such as the Wnt signaling and calcium signaling pathways in murine models. Thus, these pieces of evidence indicate a potential influence of miR-206-3p on processes like bone remodeling and mineralization, both crucial for maintaining bone strength.

Interestingly, distinctive miRNA signatures were noted between our two NGS experiments, despite employing the same T1D mouse model in both independent cohorts. To validate the miRNAs identified in the initial NGS profiling, qPCR was conducted on distinct sets of bone samples. Nevertheless, confirmation of the expression levels for all miRNAs proved challenging with no discernible trend aligning with the NGS findings. Even though we can acknowledge the different miRNA library preparations used that may have contributed to differential outcomes, the lack of reproducible results between the two NGS experiments and the qPCR validation in general is concerning as it shows that even small differences in experimental setups can significantly affect the outcome. This would imply that despite miRNAs being known as stable tools, a standardization in miRNA procedures including collection, storage, isolation, measurements etc. is necessary to identify true signatures that could be used as biomarkers (31). Another factor that may complicate the use of miRNA signatures as biomarkers is their often-minor regulation, suggesting that a substantial number of replicates may be essential to replicate findings. Overall, these data argue for the necessity to better replicate miRNA findings, especially those resulting from NGS analyses.

Finally, by examining the expression patterns of dysregulated miRNAs across various human tissues using data from the miRNA Tissue Atlas database, we found intriguing insights. Despite their dysregulation in mouse bone tissue, miRNAs such as hsa-miR-451a, hsa-miR-21a-5p, and hsa-miR-133a-3p exhibited high expression levels across multiple human tissues, suggesting potential roles beyond bone physiology. Conversely, hsa-miR-136-3p, while generally low in expression across tissues, displayed predominant expression in human bone tissue, suggesting its specific relevance to bone health. Furthermore, our gene network analysis using the miRPathDB v2.0 database revealed significant associations between dysregulated miRNAs and key signaling pathways implicated in bone metabolism, including TGF-beta, PI3-Akt signaling, osteoclast differentiation, and adherens junction signaling pathways. Especially, genes targeted by dysregulated miRNAs, such as SMAD4, MAPK1, MYC, TGFBR2, and AKT1, were identified multiple times within these pathways, highlighting their potential importance in the pathophysiology of bone loss in T1D. For instance, hsa-miR-21a-5p is extensively implicated in the TGF-beta and PI3-Akt signaling pathways, targeting critical genes such as BMPR2, TGFBR2 and AKT2, which regulate osteoblast activity, matrix production, and cell survival. Similarly, hsa-miR-133a-3p, linked to the PI3K-Akt and calcium signaling pathways, targets IGF1 and CACNA1C, emphasizing its role in modulating osteoclast differentiation and bone resorption. On the other hand, hsa-miR-451a, a key player in the PI3K-Akt and TNF signaling pathways, influences genes such as MAPK1, MYC and AKT1, underscoring its involvement in inflammation and cell proliferation within the bone microenvironment. Notably, hsa-miR-19a-3p impacts multiple pathways, including TGF-beta and Wnt signaling, targeting genes such as SMAD4 and MYC, which are pivotal for bone remodeling and repair. Lastly, hsa-miR-144-5p, through its regulation of SMAD4 and ROCK1 in the adherens junction and regulation of actin cytoskeleton pathways, may influence cytoskeletal dynamics and cell adhesion in bone tissue.

While this study provides valuable insights into miRNA regulation in T1D, it does have certain limitations. Firstly, the restriction to male mice restricts the generalizability of the results to both sexes. Secondly, while the characterization of STZ-induced T1D demonstrates a robust phenotype characterized by hyperglycemia and significant bone loss compared to non-diabetic mice, it is important to acknowledge that the utilization of only three samples per group for each NGS analysis may have been insufficient to identify genuine miRNA targets associated with bone loss in T1D. Next to the costs, the challenge of obtaining adequate bone RNA quality in our mouse experiments for NGS is why a limited number of bone samples was used. Thirdly, our study employed two distinct library preparation methods for our two NGS analyses, which may have influenced miRNA expressions and could therefore account for the discrepant results observed between NGS sequencings. These discrepancies in miRNA expression align with previous investigations evaluating miRNA expressions using four different library preparation protocols (19), as well as variations in mice, albeit less pronounced than in human samples where multiple factors such as lifestyle and family history can contribute to genetic differences. Although the use of two different library preparation methods in this study did not result in the same miRNA profile, it does underscore the inconsistent findings observed among different research institutes worldwide, given the lack of standardized guidelines for identifying miRNA targets associated with T1D-induced bone loss, or any other diseases (32).

Despite these limitations, this study possesses several strengths. We utilized a well-established mouse model of T1D, providing a reliable basis for exploratory research in miRNA profiling in T1D-induced bone loss. Our study marks the first to employ an unbiased approach to miRNA profiling in serum and bone samples from a T1D mouse model. The rationale behind conducting exploratory miRNA profiling in both serum and bone was to determine if and how well the systemic and local expression of miRNAs overlap. Finally, the experiment was conducted twice for NGS profiling in serum and bone mouse samples, providing insights into the reproducibility and sensitivity of miRNA expression.

In conclusion, our investigation presents novel findings regarding the identification of miRNA signatures in the serum and bone tissue of mice with T1D-induced bone loss employing an unbiased approach through NGS. Our study demonstrates a minor overlap in miRNA signatures between serum and bone tissues. Importantly, the utilization of independent cohorts and distinct library preparation protocols revealed varying miRNA dysregulation patterns linked to T1D-related bone disease. Hence, future research should include multiple cohorts to consistently identify and validate dysregulated miRNAs associated with T1D-induced bone disease.

## Data Availability

The datasets presented in this study can be found in online repositories. The names of the repository/repositories and accession number(s) can be found in the article/supplementary material.
